# m6A-ELISA, a simple method for quantifying *N6*-methyladenosine from mRNA populations

**DOI:** 10.1261/rna.079554.122

**Published:** 2023-05

**Authors:** Imke Ensinck, Theodora Sideri, Miha Modic, Charlotte Capitanchik, Claudia Vivori, Patrick Toolan-Kerr, Folkert J. van Werven

**Affiliations:** 1 The Francis Crick Institute, London NW1 1AT, United Kingdom; 2 Dementia Research Institute at KCL, London SE5 9RX, United Kingdom; 3 National Institute of Chemistry, SI-1001 Ljubljana, Slovenia

**Keywords:** ELISA, m6A, mESC, yeast

## Abstract

*N6*-methyladenosine (m6A) is a widely studied and abundant RNA modification. The m6A mark regulates the fate of RNAs in various ways, which in turn drives changes in cell physiology, development, and disease pathology. Over the last decade, numerous methods have been developed to map and quantify m6A sites genome-wide through deep sequencing. Alternatively, m6A levels can be quantified from a population of RNAs using techniques such as liquid chromatography-mass spectrometry or thin layer chromatography. However, many methods for quantifying m6A levels involve extensive protocols and specialized data analysis, and often only a few samples can be handled in a single experiment. Here, we developed a simple method for determining relative m6A levels in mRNA populations from various sources based on an enzyme-linked immunosorbent-based assay (m6A-ELISA). We have optimized various steps of m6A-ELISA, such as sample preparation and the background signal resulting from the primary antibody. We validated the method using mRNA populations from budding yeast and mouse embryonic stem cells. The full protocol takes less than a day, requiring only 25 ng of mRNA. The m6A-ELISA protocol is quick, cost-effective, and scalable, making it a valuable tool for determining relative m6A levels in samples from various sources that could be adapted to detect other mRNA modifications.

## INTRODUCTION

Epitranscriptomics, the study of post-transcriptional base modifications of RNAs, has been an emerging field of study for the last decade. Among all RNA modifications, *N6*-methyladenosine (m6A) is one of the most widespread and widely studied. Writer and reader proteins of the m6A RNA modification exert numerous functions in controlling the fate of mRNAs in eukaryotes, and play critical roles in development, differentiation and disease pathology (Zaccara et al. 2019; Yang et al. 2020; He and He 2021). The levels of m6A can vary between species, cell types, and conditions (Schwartz et al. 2013a; Roignant and Soller 2017; Yang et al. 2018). Hence, techniques for measuring m6A levels are essential for providing insights on the abundance and dynamics of m6A containing RNAs.

Various methods have been developed to measure m6A from mRNA populations, within single transcripts, and at nucleotide resolution (Linder et al. 2015; Bodi and Fray 2017; Garcia-Campos et al. 2019; Dierks et al. 2021; Leger et al. 2021; Mirza et al. 2022). Each of these has their purpose in helping to understand the various aspects of m6A biology. Currently only a few techniques are available for determining m6A levels in RNA populations. These include thin layer chromatography (TLC), m6A RNA dot blot, and mass-spectrometry (MS) of RNA fragmented into nucleosides (Bodi and Fray 2017; Nagarajan et al. 2019; Mathur et al. 2021). Techniques such as TLC and m6A RNA dot blot are relatively time consuming and low throughput. Additionally, measuring RNA modifications by MS often requires access to specialized instruments and specialized training. Therefore, simple and rapid techniques for measuring m6A levels in RNA samples would be useful for m6A researchers and the epitranscriptomics field as a whole.

Here we present an indirect enzyme-linked immunosorbent assay for the detection of m6A (m6A-ELISA), a method for measuring relative changes in m6A levels across mRNA samples. We optimized several steps in the protocol to obtain a high signal-to-noise ratio using yeast mRNAs. Furthermore, we show that the method can detect dynamic changes in m6A levels in yeast and in mouse embryonic stem cells (ESCs). The m6A-ELISA protocol is simple, cost efficient, and can potentially be adopted for studying other RNA modifications.

## RESULTS AND DISCUSSION

### Optimization of signal-to-noise for m6A-ELISA

To measure m6A levels within an RNA population, we set out to develop a detection method based on ELISA (Lin 2015). In short, mRNA is bound directly to a microplate using a commercially available nucleic acid microplate binding solution. The bound mRNA is then incubated with a primary anti-m6A antibody. This incubation is followed by the addition of a secondary, HRP-coupled antibody which allows a colorimetric readout using generic ELISA substrates.

**FIGURE 1.**
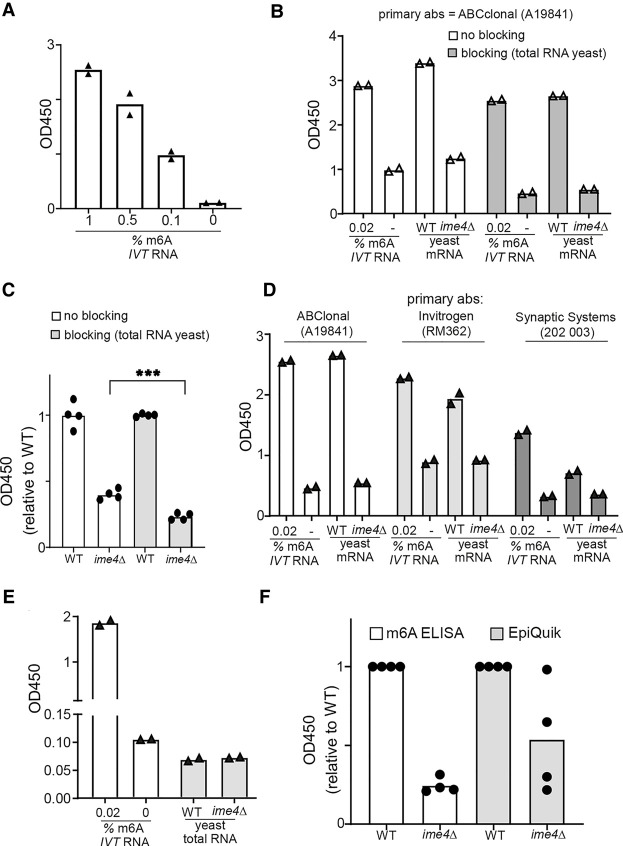
Optimization of signal-to-noise for m6A-ELISA. (*A*) Testing of m6A antibody specificity. Various ratios of in vitro transcribed (IVT) RNA unmodified adenosine (*A*), and IVT m6A containing RNA (m6A) were used (1%, 0.5%, 0.1%, or 0% m6A IVT RNA). In total, 50 ng IVT RNA was used for each sample. Displayed are the mean and individual OD_450_ values of *n* = 2 technical replicates from a representative experiment. (*B*) Primary antibody and blocking reagent testing. Primary antibody (ABClonal-A19841) was incubated with or without 0.5 µg/mL of total RNA to reduce background binding to unmodified RNA. We used 50 ng in vitro transcribed (IVT) RNA generated with unmodified adenosine (*A*), and 0.01 ng of IVT m6A containing RNA (m6A). Additionally, we used poly(A)-selected mRNA samples from wild-type (WT) (FW1511) diploid cells and cells harboring gene deletion in *IME4* (*ime4*Δ) (FW7030) that were induced to enter meiosis. Displayed are the mean and individual OD_450_ values of *n* = 2 technical replicates. (*C*) Similar analysis as in *B*. We used 50 ng poly(A)-selected mRNA samples from WT and *ime4*Δ cells that were induced to enter meiosis, and normalized samples to the WT signal. Displayed are *n* = 4 technical replicates across *n* = 2 independent experiments (unpaired *t*-test of *P* = 0.0004). (*D*) Primary antibody testing of samples using m6A-ELISA. Two m6A antibodies (Invitrogen-RM362 and Synaptic systems 202003) were tested in the m6A-ELISA. Displayed are the mean and individual OD_450_ values of *n* = 2 technical replicates from a representative experiment. (*E*) Similar analysis as in *A*, except that isolated total RNA was used for the analysis for WT and *ime4*Δ cells induced to enter meiosis. Displayed are the mean and individual OD_450_ values of *n* = 2 technical replicates from a representative experiment. (*F*) Comparison of m6A with a commercial m6A ELISA (EpiQuik). Like in *A*, the m6A signal for WT and *ime4*Δ cells were compared. For each experiment, the *ime4*Δ OD_450_ signals were normalized to WT OD_450_ signal (set to 1). The mean and individual values of *n* = 4 independent experiments are shown.

To optimize the m6A-ELISA, we considered variables that could influence the signal-to-noise ratio. These variables included background signal from nonspecific binding by primary antibodies, blocking reagents, and the method of RNA preparation. As biological samples, we isolated mRNA from diploid budding yeast cells in the early phase of the meiotic program. In this stage, m6A is abundant because the m6A writer complex, including the yeast METLL3 orthologue Ime4, is expressed and active (Clancy et al. 2002; Agarwala et al. 2012). Importantly, diploid cells harboring an *IME4* gene deletion display no detectable levels of m6A thus forming the ideal negative control for optimizing the m6A-ELISA protocol (Schwartz et al. 2013a). In yeast meiosis, m6A levels are at most 0.1% (Agarwala et al. 2012; Varier et al. 2022). First, we assessed whether m6A levels could be detected using the ABClonal-A19841 antibody (Fig. 1A,B; Supplemental Fig. 1A). We optimized m6A antibody concentrations for high signal-to-noise ratios by measuring the raw ELISA signals (OD_450_) and by comparing mRNAs isolated from WT yeast entering meiosis to *ime4*Δ (Fig. 1B). Additionally, we tested antibody specificity with in vitro transcribed (IVT) RNAs that contain either only unmodified adenosines or only m6A modified adenosines, which we mixed in different ratios. When we used 100%, 10%, or 1% m6A IVT RNA, we obtained a high signal compared to the unmodified IVT RNA only (more than 10-fold over background) indicating that the m6A antibody is highly specific (Supplemental Fig. 1A). However, at these concentrations, the m6A IVT signal was saturated. Lowering the concentration from 1% to 0.1% showed a corresponding decrease in signal, indicating that the signal was not saturated in this range (Fig. 1A). M6A ELISA with yeast mRNAs showed a threefold enrichment (WT vs. *ime4*Δ), which was comparable to the signal-to-noise ratio obtained with only 0.02% IVT m6A RNA (mixed with 99.98% IVT unmodified RNA) vs. IVT unmodified RNA (Fig. 1B).

Nonspecific binding of anti-m6A antibodies to RNA has been detected when *ime4*Δ was used as the negative control in anti-m6A mRNA pulldown followed by sequencing (Schwartz et al. 2013a). To reduce the unspecific background binding of the primary antibody, we used total RNA from *ime4*Δ cells as a blocking reagent when we incubated the primary antibody with samples (Fig. 1B,C). The background binding to IVT unmodified RNA was reduced when competing total RNA was added (Fig. 1B). The background signal was dependent on the presence of RNA bound to wells and not due to unspecific binding of the primary antibody alone (Supplemental Fig. 1B). We also tested yeast tRNA as a possible blocking reagent, but this did not result in lower background binding (Supplemental Fig. 1B). The blocking with total RNA also significantly improved the m6A ELISA signal-to-noise with yeast mRNAs (WT vs. *ime4*Δ) (Fig. 1C). Other anti-m6A antibodies (Invitrogen-RM362 and Synaptic systems 202003) under the same conditions showed enrichment for both the IVT m6A RNA and yeast mRNAs compared to unmodified RNA controls (in vitro transcribed unmodified RNAs, and *ime4*Δ) (Fig. 1D). However, the highest signal-to-noise ratio was observed with the A19841 antibody for both in vitro transcribed RNA and yeast mRNA (Fig. 1B,C). This prompted us to use the A19841 antibody for the subsequent experiments.

M6A occurs primarily on mRNAs, but it is also present on ribosomal RNA (Maden 1986; Zaccara et al. 2019; Ignatova et al. 2020; Leismann et al. 2020; Mirza et al. 2022). Hence, we typically perform m6A based assays on polyadenylated [poly(A)] purified mRNAs. However, some commercially available m6A assays claim that total RNA can used for the analysis (see: Materials and Methods). Moreover, there is no evidence that yeast ribosomal RNA is modified by m6A. This prompted us to test whether total RNA isolated from yeast is suitable for detecting m6A on mRNAs by m6A-ELISA. We observed no enrichment in m6A signal in WT total RNA samples compared to *ime4*Δ, likely because m6A levels were too low to be detected (Fig. 1E). Total RNA m6A signal was comparable to the IVT RNA with unmodified A, suggesting that there is no detectable m6A on yeast total RNA. Increasing the amount of total RNA did not improve the ability to detect m6A levels (Supplemental Fig. 1C). Also, when we used a commercial m6A ELISA assay (EpiQuik) we also observed no difference between WT compared to *ime4*Δ (Supplemental Fig. 1D). We conclude that m6A-ELISA works on poly(A)-selected mRNAs but does not detect m6A on isolated total RNA.

Next, we compared the m6A ELISA signal to noise directly with a commercial m6A ELISA assay (EpiQuik). We performed multiple independent assays using both protocols. We found that our m6A-ELISA raw values showed comparable signal to noise ratios compared to two of the replicates from the EpiQuik assay (Fig. 1F). However, two of the replicates of the EpiQuik assay showed little difference between WT and *ime4*Δ. We conclude that our m6A ELISA assay performed at least as good as the EpiQuik assay (Fig. 1F).

### m6A ELISA quantification compared to m6A MS

**FIGURE 2.**
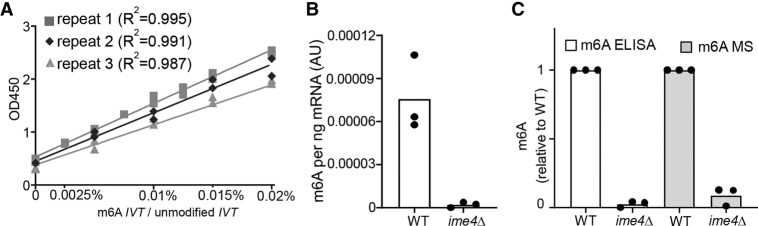
Validation of m6A-ELISA. (*A*) Standards generated from serial dilutions and mixing of IVT RNA with m6A modified adenosine and unmodified adenosine. Each standard dilution contained 50 ng IVT unmodified RNA with different quantities (0–10 pg) of m6A modified IVT RNA. The mean and individual values of *n* = 3 independent experiments are shown. (*B*) Quantification of m6A levels using m6A ELISA. mRNA from WT and *ime4*Δ cells induced to enter meiosis was used for the analysis. A dilution series described in *A* was used to transform the OD_450_ values to m6A signal (AU) per ng mRNA. The mean and individual values of *n* = 3 independent experiments are shown. (*C*) Comparison of m6A ELISA and m6A MS using mRNA from WT and *ime4*Δ cells. The data from the m6A MS data was obtained from Varier et al. (2022). To make a direct comparison, for each WT vs. *ime4*Δ experiment the WT signal was set to 1. The mean and individual values of *n* = 3 independent experiments are shown.

So far, our analysis was focused on improving the signal to noise ratios as determined by the raw m6A ELISA values (OD_450_). Next, we introduced a standard for the m6A-ELISA by making serial dilutions of IVT m6A labeled RNAs, which we used to normalize the raw values. Given that the antibody has an affinity for unmodified RNA, albeit much weaker compared to m6A-containing RNA, we kept the amount of RNA for each sample constant. Hence, we generated serial dilutions using IVT unmodified RNA mixed with various amounts of IVT m6A RNA, typically 50 ng in total. We tested the standard curve linearity and reproducibility from three independent experiments. We found that the standards gave linear slopes (*R*^2^ = 0.99) and were reproducible (Fig. 2A). We used the standard the curve to quantify the m6A levels in WT mRNA samples compared to *ime4*Δ in multiple independent assays. The standard curve corrected for the background signal, and we observed more than 30-fold signal to noise ratio (Fig. 2B). The signal-to-noise ratios obtained with standard curve corrected sample quantifications were comparable to m6A measurements obtained using LC-MS (Fig. 2C; Varier et al. 2022).

### Critical steps of m6A-ELISA protocol

**FIGURE 3.**
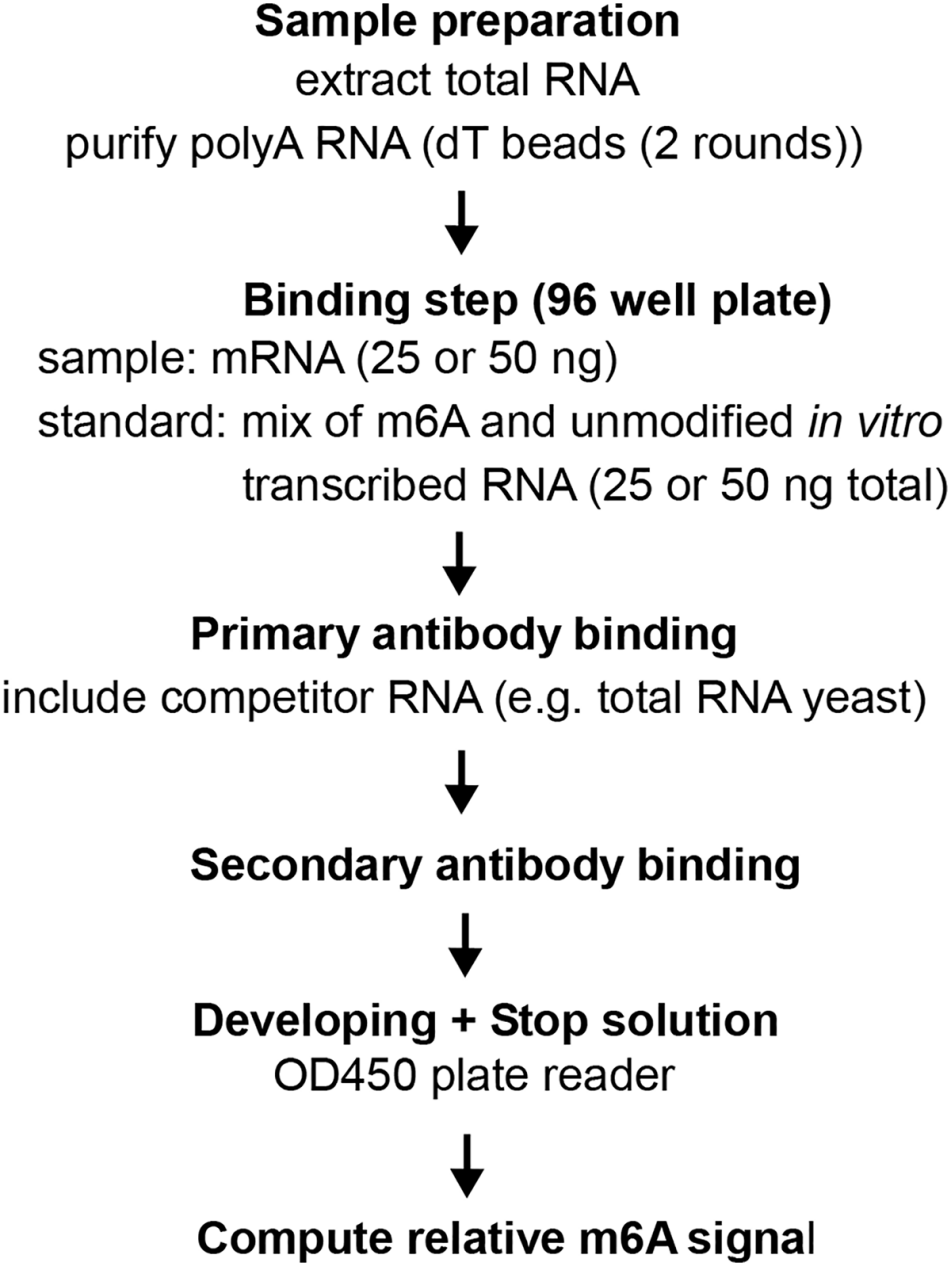
Overview of the m6A-ELISA protocol.

The critical steps of the m6A-ELISA protocol are summarized in Figure 3. First, total RNA is purified from cells and mRNA is subsequently isolated by two rounds of poly(A) purification. The minimum amount we tested is two times 25 ng mRNA per sample, which can be recovered from 5 µg of total RNA. The purified mRNAs are quantified and incubated with binding solution to allow RNA binding to wells. We typically include positive and negative controls (WT and *ime4*Δ) and standards generated from IVT RNAs. The next steps include primary antibody incubation plus blocking RNA, washes, secondary antibody incubation, washes, and incubation with substrate solution. Noteworthy, primary antibody concentrations should be tested empirically with standards and on control samples when using a new batch of antibodies. Stop solution is added after the positive controls have developed a medium blue color, after approximately 10 to 30 min. For the analysis, we compute a standard curve from the IVT m6A RNA serial dilutions, which we use to normalize the absorbance signal of samples. The m6A-ELISA assay has the potential to give an absolute quantitative signal if the number of As in the standards are well defined and are spaced within the transcript for antibody accessibility. However, we did not consider this for the standard we used. In addition, while the relative differences and trends among samples were reproducible, we observed that samples and standards can give some level of variability between different ELISA experiments. Therefore, it is preferred to include samples that are directly compared to each other in the same m6A-ELISA experiment. Ideally, positive and negative control samples should be included, for example, WT and *ime4*Δ. The m6A-ELISA protocol thus allows for rapid measurement of relative m6A abundance in mRNA samples.

### m6A-ELISA can detect incremental changes in m6A levels

**FIGURE 4.**
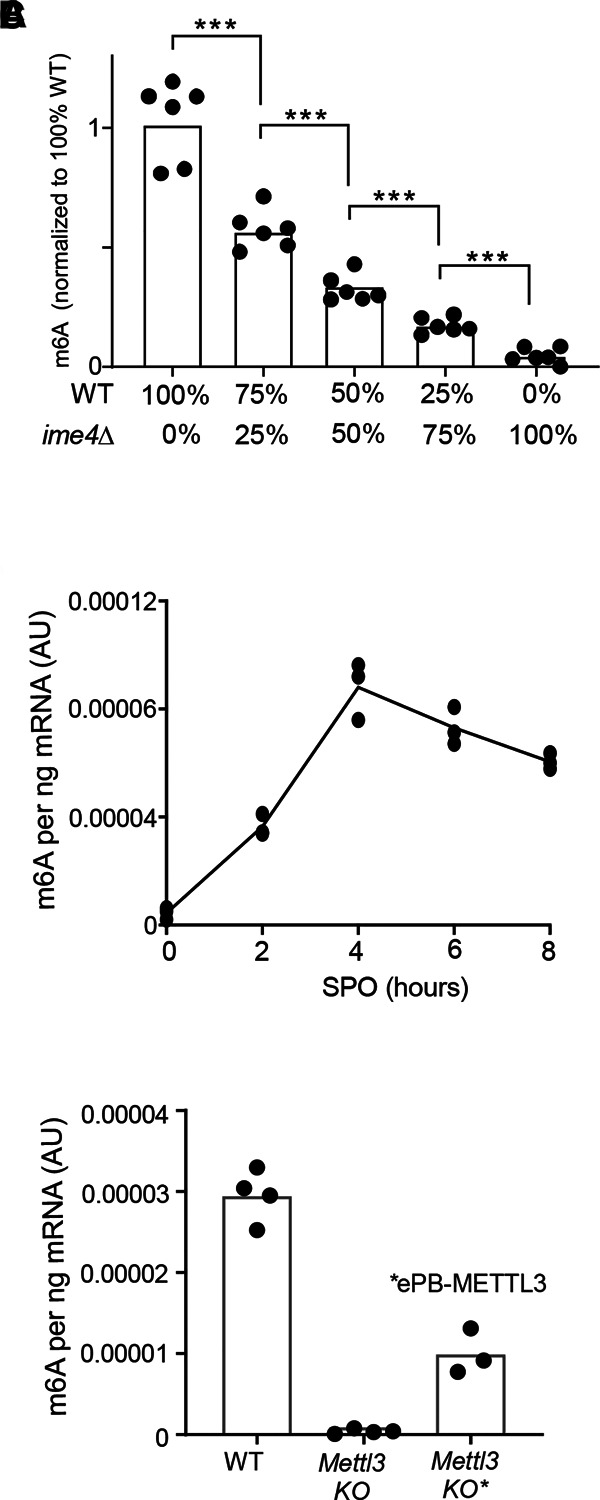
m6A-ELISA detects incremental changes in m6A. (*A*) Quantification of m6A RNA levels in WT and *ime4*Δ. WT and *ime4*Δ samples were mixed in various proportions: 100% vs. 0%, 75% vs. 25%, 50% vs. 50%, 25% vs. 75%, and 0% vs. 100% (WT vs *ime4*Δ). Signals were normalized to standard curve, and subsequently the WT signal was set to 100%. Unpaired *t*-test, *n* = 6 technical replicates in *n* = 2 independent experiments, each step *P* < 0.0005. (*B*) m6A deposition throughout yeast meiosis. Samples were taken at the indicated time points, and m6A levels were determined. Signals were normalized to the standard curve. The mean and individual values of *n* = 3 replicates are shown. (*C*) m6A levels determined by m6A-ELISA using mRNA isolated from WT and *Mettl3* KO mESC, and *Mettl3* KO* mESC line. (*) Also contains a copy of human METTL3 under control of a doxycycline inducible promoter. An amount of 25 ng of RNA was used for the m6A-ELISA. The mean and individual values for the WT and Mettl3 KO *n* = 4 replicates are shown of two mESC cultures, and *Mettl3* KO* *n* = 3 replicates.

Next, we examined whether the m6A-ELISA method can detect relatively small changes in m6A levels, and thus can be used to determine quantitative differences. We mixed different ratios of yeast WT and *ime4*Δ mRNAs. The signal increased with the relative amount of m6A-containing wild type mRNA (Fig. 4A). As expected, when we used mRNAs from *ime4*Δ cells only, the normalized signal was close to background levels. Additionally, when we made 25% incremental increases in WT vs. *ime4*Δ mRNA ratios, the m6A signal increased proportionately. Thus, the m6A-ELISA method can detect 25% reduction in m6A levels, as each 25% decrease was significant.

We also determined m6A dynamics in yeast. In budding yeast, m6A rapidly increases in early stages of meiosis and subsequently decreases as cells progress further and form spores (Agarwala et al. 2012; Schwartz et al. 2013b). To test whether m6A-ELISA can detect the dynamics of m6A deposition, we took mRNA samples throughout meiosis. We found that m6A levels increased and decreased in line with what has been described previously, indicating that m6A-ELISA is suitable for detecting incremental changes in m6A levels (Fig. 4B; Agarwala et al. 2012; Schwartz et al. 2013a).

Lastly, we measured relative m6A levels in WT mESCs and a *Mettl3* knockout mESC line (Fig. 4C). M6A levels were reduced over 50-fold in the *Mettl3* knockout cell line, which is consistent with the findings that METTL3 is the major m6A methyltransferase for mRNAs in mammals (Poh et al. 2021). Furthermore, we found that low technical variability could be achieved when using as little as 25 ng mammalian mRNA (Fig. 4C). We also included a *Mettl3* knockout cell line that expressed the human METTL3 transgene from a doxycycline inducible promoter. This cell line showed leaky expression of METTL3 (Supplemental Fig. 3A,B). The leaky expression of METTL3 observed in the absence of doxycycline, resulted in a 20-fold increase of m6A levels compared to the *Mettl3* knockout, but m6A levels were still about fourfold lower compared to WT. We conclude that m6A-ELISA can detect changes in m6A levels in purified mRNAs both in yeast and mammals.

In summary, we have developed an m6A-ELISA assay for quick and cost-effective detection of relative m6A levels from mRNA samples. For the m6A-ELISA protocol, we identified and optimized critical steps that improved the signal-to-noise ratio for detection. Noteworthy, we recently applied the m6A-ELISA to measure the m6A relative mRNA decay rate (Varier et al. 2022). The m6A-ELISA method could also be adapted to other mRNA modifications, provided specific antibodies are available. The m6A-ELISA is a quick, easy, and accessible detection method for global m6A detection. We propose that m6A-ELISA can be used to rapidly measure relative m6A levels across different cell types or mutants, as well as to determine the dynamic changes of m6A levels over time.

## MATERIALS AND METHODS

### Yeast strains and grown conditions

Entry into meiosis was induced in the yeast SK1 diploid cells as previously described, following a standard protocol (Moretto et al. 2021). The wild-type and *ime4*Δ strains (FW1511 and FW7030) were previously described (Varier et al. 2022). In short, cells were grown at 30°C to saturation in YPD [1% (w/v) yeast extract, 2% (w/v) peptone, 2% (w/v) glucose supplemented with 24 µg/mL uracil and 12 µg/mL adenine], then diluted at OD_600_ = 0.4 to presporulation medium BYTA [1.0% (w/v) yeast extract, 2.0% (w/v) bacto tryptone, 1.0% (w/v) potassium acetate, 50 mM potassium phthalate] and grown for 16–18 h, and finally transferred at OD_600_ = 1.8 to sporulation medium SPO [0.3% (w/v) potassium acetate and 0.02% (w/v) raffinose]. Cell pellets for RNA extraction were collected 4 h after transfer to the sporulation medium.

### Mouse embryonic stem cell (mESC) culture

mESCs were maintained in 2i + LIF conditions mESC and were cultured in N2B27-based medium in 2i + LIF conditions (1000 U/mL LIF [ESGRO ESG1107, Merck], 1 µM PD0325901 [04-0006, Stemgent], and 3 µM CHIR99021 [1386 Axon Medchem]) (Modic et al. 2019). Cells were passaged using StemPro Accutase (A1110501, Thermo Fisher Scientific) and grown on 0.1% gelatin (G1393, Sigma-Aldrich)-coated plates.

### Generation of the CRISPR/Cas9 genome engineered *Mettl3* KO mESC line

To generate Mettl3 knockout cells, we used single gRNAs with target sites within the sixth Mettl3 exon to introduce an out-of-frame point mutation. gRNA oligos were cloned into the SpCas9-T2A-PuroR/gRNA vector (px459) via cut-ligation (gRNA sequence TTGTGATGGCTGACCCACCT). IDG3.2 mESCs were transfected with an equimolar amount of each gRNA vector. Two days after transfection, cells were plated at clonal density and subjected to a transient puromycin selection (1 µg/mL) for 40 h. Colonies were picked 6 d after transfection, and PCR primers were used to identify clones in which the sixth exon had been modified. This was confirmed with Sanger sequencing, and loss of METTL3 expression was assessed by western blot (Supplemental Fig. S3A). The western blot was probed for METTL3 (Abcam, ab195352), Mettl14 (Abcam, ab264408), and GAPDH (Abcam, ab8245).

### Generation of Mettl3 KO plus inducible FLAG-METTL3 mESC line

To generate the inducible PiggyBac donor vector with an amino-terminal FLAG tag fused to METTL3, we first synthesized gBlock (IDT) containing the BamHI-1XFLAG-METTL3-BamHI sequence. This fragment was then cloned into the BamHI entry site in the enhanced piggyBac transposable vector epB-Bsd-TT via cut-ligation to generate the vector, ePB-1xFLAG-METTL3 (Rosa et al. 2014).

Mettl3 KO mESCs were transfected with an equimolar amount of ePB-1xFLAG-METTL3 and piggyBac transposase (Rosa et al. 2014). Two days after transfection, cells were plated at clonal density and subjected to a transient puromycin selection (1 µg/mL) for 7 d. Colonies were picked 12 d after transfection, and western blot against endogenous METTL3 (Abcam, ab195352) was performed to identify clones which maintain comparable METTL3 expression between WT mESC cells and the ePB-METTL3 mESC line that was generated from parental Mettl3 KO mESCs. For experiments involving the ePB-METT3 ESC line, all inductions were performed using 100 ng/mL doxycycline (Sigma-Aldrich). A western blot of induced and uninduced cells, probed for METTL3 (Abcam, ab195352) and histone H3 (Abcam, ab1791), is shown in Supplemental Figure 3B. For the m6A-ELISA, mESC lines were cultured in 2i + LIF conditions on gelatin 0.1%-coated plates. Cells were harvested with Accutase (A1110501, Life Technologies).

### RNA extraction

Total RNA was extracted from frozen yeast pellets as previously described, using Acid Phenol:Chloroform pH 4.5 and Tris-EDTA-SDS (TES) buffer (0.01 M Tris-HCl pH 7.5, 0.01 M EDTA, 0.5% w/v SDS). The mixture was incubated at 65°C for 45 min (1400RPM), transferred to ice, and centrifuged at 16,000*g* 4°C for 10 min. The aqueous phase was transferred to ethanol with 0.3 M sodium acetate and RNA was precipitated at −20°C overnight. After centrifugation (16,000*g*, 30 min) and washing with 80% (v/v) ethanol solution, dried RNA pellets were resuspended in RNase-free water (Ambion). Samples were further treated with rDNase (740.963, Macherey-Nagel) and column purified (cat. no. 740.948, Macherey-Nagel), following manufacturer's protocols. RNA from mESCs was extracted using the RNeasy Mini Kit (74104, Qiagen) with on-column rDNase digestion (79254, Qiagen) according to manufacturer's protocol.

### Poly(A) selection

Total RNA was enriched for poly(A)^+^ RNA using Oligo(dT)25 Dynabeads (Invitrogen 61005) according to manufacturer's protocol. Two consecutive rounds of purification were performed, an initial round with 75 µg total RNA and 200 µL bead solution, and a second round with the eluted RNA and 40 µL bead solution.

### In vitro transcription for generating standards

Standards were generated using the MEGAscript T7 Transcription Kit (Invitrogen AM1333), following the manufacturer's instructions and using the included control template. Reactions either used the included ATP nucleotide solution, or *N6*-Methyl-ATP (Jena Bioscience NU-1101L) at the same concentration. For a 50 ng standard, 50 ng A containing RNA was supplemented with 0–15 pg of m6A containing RNA.

### ELISA assay

RNA concentrations were determined using the Qubit RNA High Sensitivity (HS) Kit. An amount of 90 µL of binding solution (ab156917) was added to a clear 96-well plate (ab210903) and mixed with the desired amount of mRNA sample (typically 50 ng or 25 ng). Standards containing the same quantity of in vitro transcribed RNA were used. The plate was incubated at 37°C for 2 h, each well was washed four times with PBST (0.1%), and 100 µL of primary antibody solution was added and incubated for 1 h at room temperature (1:10000 Abclonal A19841 including 0.5 µg/mL competing *ime4*Δ yeast total RNA). Each well was then washed four times, incubated with secondary antibody solution for 30 min at room temperature (1:5000 ab205718), and washed five times. An amount of 100 µL TMB ELISA Substrate (Fast Kinetic Rate) (ab171524) was added, and the samples were developed for up to 30 min. The reaction was stopped by the addition of 100 µL stop solution (ab171529). The absorbance was read at 450 nm using a Tecan Sunrise plate reader and Magellan software. A detailed stepwise protocol is included in Supplemental File 1. The m6A RNA Methylation Quantification Kit from EpiQuik (EpiGentek, P-9005) was performed according to manufacturer's protocol, except 100 ng mRNA was used instead of the recommended total RNA in designated samples.

### M6A quantification by LC-MS

Data from the m6A LC-MS experiment were previously described in Varier et al. (2022).

## SUPPLEMENTAL MATERIAL

Supplemental material is available for this article.

## Supplementary Material

Supplemental Material

## Figures and Tables

**Imke Ensinck:**
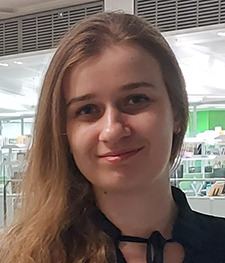


## MEET THE FIRST AUTHOR

***Meet the First Author(s)* is an editorial feature within *RNA*, in which the first author(s) of research-based papers in each issue have the opportunity to introduce themselves and their work to readers of *RNA* and the RNA research community. Imke Ensinck is the first author of this paper, “m6A-ELISA, a simple method for quantifying *N6*-methyladenosine from mRNA populations.” Imke is a PhD student in the laboratory of Folkert van Werven at the Francis Crick Institute in London. She studies the protein complex involved in the *N6*-methyladenosine (m6A) mRNA modification in budding yeast.**

**What are the major results described in your paper and how do they impact this branch of the field?**

In this paper, we present a simple and cost-effective method for determining *N6*-methyladenosine (m6A) modification levels in mRNA samples, based on an enzyme-linked immunosorbent-based assay (m6A-ELISA). Many techniques to determine m6A levels in mRNA populations are labor intensive or require specialized equipment. This technique is accessible, and would allow researchers to quickly and cheaply determine the relative m6A levels across different cell types and mutants, which can then be analyzed further.

**What led you to study RNA or this aspect of RNA science?**

As my PhD research focuses on the m6A deposition machinery in budding yeast, I needed an accessible and cost-effective way to test the relative m6A modification levels in many mutants. After mixed experiences with other techniques and commercially available options, we developed m6A-ELISA to fit our needs. As the method was very helpful to us, we decided to share it so that hopefully it can benefit other researchers as well!

**During the course of these experiments, were there any surprising results or particular difficulties that altered your thinking and subsequent focus?**

m6A-ELISA relies on antibodies to detect m6A. We were therefore worried that the nonspecific binding of these antibodies to RNA would strongly affect our results. However, we found that this nonspecific binding can be significantly reduced by adding competing m6A-free RNA in the antibody mixture, similarly to how protein mixtures are used to block antibodies for protein detection! It was a straightforward approach, yet very impactful on the signal-to-noise ratio of our data.

**What are some of the landmark moments that provoked your interest in science or your development as a scientist?**

I have been very lucky to have had great supervisors during all my research projects, in particular my internship supervisor Laura Ledesma García, and my PhD supervisor Folkert van Werven. They allowed me to pursue my own scientific ideas, while still being very involved as supervisors. I credit a lot of my improvements as a scientist to their supervision styles, and I would encourage supervisors to allow their students similar freedoms so they can grow to be the best scientists they can be!
