# Supplementary material for: m6A-ELISA, a simple method for quantifying N6-methyladenosine from mRNA populations
Source: RNA. 2023 May;29(5):705–12. doi: 10.1261/rna.079554.122 (PMC10159001; doi:10.1261/rna.079554.122)
Supplement: Supplemental Material [file supp_29_5_705__DC1.html]

m6A-ELISA, a simple method for quantifying N6-methyladenosine from mRNA populations — Supplemental Material 

# m6A-ELISA, a simple method for quantifying *N6*-methyladenosine from mRNA populations

## Supplemental Material

- Supplemental\_Figures.pdf
- Supplemental\_File\_1.docx
