# Supplementary material for: m6A-ELISA, a simple method for quantifying N6-methyladenosine from mRNA populations
Source: RNA. 2023 May;29(5):705–12. doi: 10.1261/rna.079554.122 (PMC10159001; doi:10.1261/rna.079554.122)
Supplement: Supplemental Material [file supp_079554.122_Supplemental_Figures.pdf]

Supplementary Figure 1

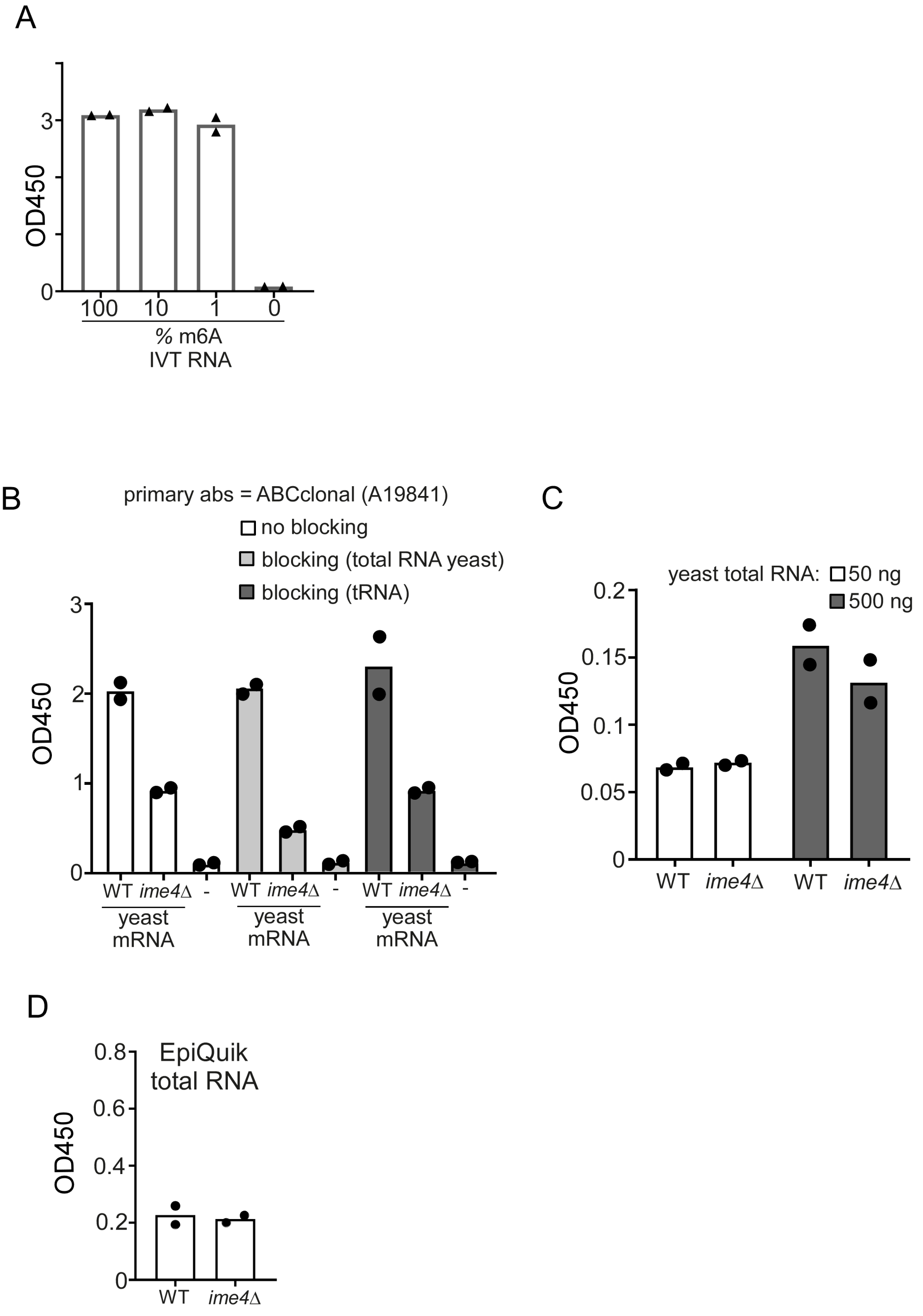

Supplementary Figure 1.
(A) Antibody specificity test. Various ratios of in vitro transcribed (IVT) RNA unmodified adenosine (A), and IVT m6A containing RNA (m6A) were used (100%, 10%, 1%, or 0% m6A IVT RNA). In total 50ng IVT RNA was used for each sample. Displayed are the mean and individual OD450 values of n=2 technical replicates from a representative experiment. **(B)** Comparison of different blocking reagents. Primary antibody (ABClonal-A19841) was incubated with no blocking RNA, with 0.5 μg/mL of total RNA or 5 μg/mL tRNAs to reduce background signal. We used polyA-selected mRNA samples from wild-type (WT) (FW1511) diploid cells and cells harbouring gene deletion in *IME4* (*ime4*Δ) (FW7030) that were induced to enter meiosis. We also determined the m6A-ELISA signal of wells with no sample (-). Displayed are the mean and individual OD450 values of n=2 technical replicates from a representative experiment. **(C)** m6A-ELISA on samples isolated from total RNA. Total RNA was used for the analysis of samples isolated from WT and *ime4*Δ cells induced to enter meiosis. 50 ng and 500 ng of total RNA was used for the analysis. Displayed are the mean and individual OD450 values of n=2 technical replicates from a representative experiment. **(D)** m6A-ELISA using the EpiQuik assay. We performed m6A-ELISA on total RNA from WT and *ime4*Δ cells. Displayed are the mean and individual OD450 values of n=2 technical replicates from a representative experiment.

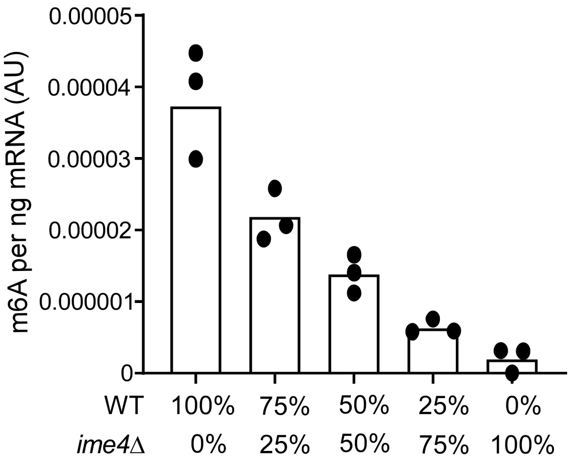

Supplementary Figure 2.

Quantification of m6A RNA levels in WT and *ime4Δ*. Signals were normalized to standard curve. WT and *ime4Δ* samples were mixed in various proportions: 100% vs 0%, 75% vs 25%, 50% vs 50%, 25% vs 75% and 0% vs 100% (WT vs *ime4Δ*). The mean and individual values of n=3 independent experiments are shown.

Supplementary Figure 3

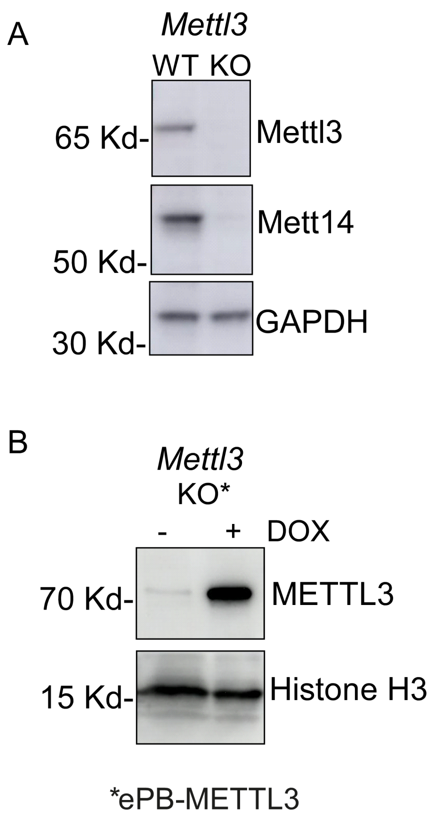

Supplementary Figure 3.

(A) Western blots of WT and Mettl3 KO mESC. Membranes were probed for METTL3 (Abcam, ab195352), Mettl14 (Abcam, ab264408), and GAPDH (Abcam, ab8245). **(B)** Western blot of WT and Mettl3 KO harbouring the human METTL3 under control of doxycycline inducible promoter mESC. Cells were grown in 2iLIF in the absence or presence of 100ng/ml doxyclyine. Membranes were probed for METTL3 (Abcam, ab195352), and histone H3 (Abcam, ab1791). *Mettl3 KO mESC line also harbouring human METTL3 under controls of a doxycycline inducible promoter.
