# Supplementary material for: m6A-ELISA, a simple method for quantifying N6-methyladenosine from mRNA populations
Source: RNA. 2023 May;29(5):705–12. doi: 10.1261/rna.079554.122 (PMC10159001; doi:10.1261/rna.079554.122)
Supplement: Supplemental Material [file supp_079554.122_Supplemental_File_1.docx]

| **Protocol for ELISA:**  1. Add 90 ul binding solution (ab156917) to each well. |
| --- |
|  |
| 2. Add 25 or 50 ng mRNA to each well. Use at least duplicates. *mRNA is obtained from total RNA by two rounds of PolyA enrichment using Oligo-dT Dynabeads. Concentrations are determined using Qubit RNA High Sensitivity kit.* |
|  |
| 3. Incubate at 37C for 2h.  4. Prepare the primary antibody solution (mRabbit A19841, 1:10000 in PBST) with 0.5 ug/mL total RNA from *ime4Δ* (7030) strain. 100uL is needed for each well. *Adding competing yeast total RNA (without m6A) to the antibody solution improves the signal to noise ratio without causing any background signal.* |
|  |
| 5. Remove binding solution with multichannel and wash each well with 4x 200uL PBST (0.1%). |
|  |
| 6. Add 100ul of primary Ab dilution (*step 4*) to each well. Make sure to remove any bubbles. Incubate for 1h at room temperature. |
|  |
| 7. Remove and wash 4x with 200uL PBST. |
|  |
| 8. Add 100uL secondary antibody solution (anti-rabbit ab205718 1:5000 in PBST). Make sure to remove any bubbles. Incubate for 30 minutes at room temperature. |
|  |
| 9. Remove and wash 5x with 200uL PBST. After final wash, make sure to tap plate onto tissue to remove as much of the PBST as possible. |
|  |
| 10. Add 100 ul developing ('fast kinetic rate' ab171524) solution. Make sure to remove any bubbles. Keep covered from light. *Optional: The development can be measured on plate reader at 650+450nm.* |
|  |
| 11. When satisfied with development, stop the plate reader and add 100uL stop solution. *Differences in signal can often be observed within a few minutes, but I found that a longer development of up to 30 minutes works well for my samples.* |
|  |
| 12. Read absorbance at 450 nm. |

**Appendix: Standard curves for ELISA.**

The standard curve contains *in vitro* transcribed mRNA without m6A (‘A’) mixed with small amounts of *in vitro* transcribed mRNA using only m6ATP and no ATP (‘m6A’). The A only control, using an equal amount of mRNA as the samples, is crucially important as it allows to correct for background binding of the antibody to RNA.

*Instead of a full standard curve, a positive (A+m6A) and negative (A only) control could be used.*

Suggested amounts:

| 50ng A + 0.015ng m6A |
| --- |
| 50ng A + 0.01 ng m6A |
| 50ng A + 0.0075 ng m6A |
| 50ng A + 0.005 ng m6A |
| 50ng A + 0.00375 ng m6A |
| 50ng A + 0.0025 ng m6A |
| 50ng A + 0.00125 ng m6A |
| 50ng A |

To make the standard curve

- Make dilutions of m6A *in vitro RNA*
- Dilute 1:10 m6A RNA down to 1ng/uL
- Dilute 1ng/uL to 0.02ng/uL (98uL H2O + 2uL stock)
- Dilute further:

| standard | uL 0.02ng/uL stock | uL H2O |
| --- | --- | --- |
| 0.015ng/uL | 15 | 5 |
| 0.01ng/uL | 10 | 10 |
| 0.0075ng/uL | 7.5 | 12.5 |
| 0.005ng/uL | 5 | 15 |
| 0.00375ng/uL | 3.75 | 16.25 |
| 0.0025ng/uL | 2.5 | 17.5 |
| 0.00125ng/uL | 1.25 | 18.75 |

- Measure concentration of A *in vitro* RNA and determine amount for 50ng
- Add 90ul binding solution to each well, then add 50ng in vitro A RNA to each well, then add 1uL of each standard

**Materials and reagents**

Materials ELISA:

- 96-Well Microplate Pack (ab210903)
- DNA Binding Microplate Solution (ab156917)
- N6-methyladenosine / m6A Rabbit mAb (A19841)
- Phosphate buffered saline (PBS) + 0.1% TWEEN-20 (9416) (PBST)
- Goat Anti-Rabbit IgG H&L (HRP) (ab205718)
- TMB ELISA Substrate (Fast Kinetic Rate) (ab171524)
- 450 nm Stop Solution for TMB Substrate (ab171529)
- TECAN Sunrise Plate Reader

Generating standards:

- MEGAscript T7 Transcription Kit-25 reactions (AM1333)
- N6-Methyl-ATP (NU-1101L)

Preparing mRNA samples from total RNA:

- Qubit™ RNA High Sensitivity (Q32855)
- Dynabeads™ Oligo(dT)25 (61005)
